# Enhancement of the Comb Filtering Selectivity Using Iterative Moving Average for Periodic Waveform and Harmonic Elimination

**DOI:** 10.1155/2018/7901502

**Published:** 2018-02-01

**Authors:** José L. Ferreira, Yan Wu, Ronald M. Aarts

**Affiliations:** ^1^Department of Electrical Engineering, Eindhoven University of Technology, P.O. Box 513, 5600 MB Eindhoven, Netherlands; ^2^Philips Research Laboratories, Eindhoven, Prof. Holstlaan 4, 5656 AE Eindhoven, Netherlands

## Abstract

A recurring problem regarding the use of conventional comb filter approaches for elimination of periodic waveforms is the degree of selectivity achieved by the filtering process. Some applications, such as the gradient artefact correction in EEG recordings during coregistered EEG-fMRI, require a highly selective comb filtering that provides effective attenuation in the stopbands and gain close to unity in the pass-bands. In this paper, we present a novel comb filtering implementation whereby the iterative filtering application of FIR moving average-based approaches is exploited in order to enhance the comb filtering selectivity. Our results indicate that the proposed approach can be used to effectively approximate the FIR moving average filter characteristics to those of an ideal filter. A cascaded implementation using the proposed approach shows to further increase the attenuation in the filter stopbands. Moreover, broadening of the bandwidth of the comb filtering stopbands around −3 dB according to the fundamental frequency of the stopband can be achieved by the novel method, which constitutes an important characteristic to account for broadening of the harmonic gradient artefact spectral lines. In parallel, the proposed filtering implementation can also be used to design a novel notch filtering approach with enhanced selectivity as well.

## 1. Introduction

**Figure 1:**
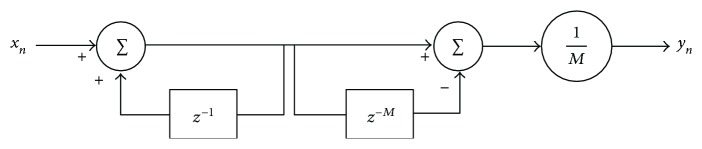
Discrete time realisation of the moving average filter described in (2).

In biomedical signal processing and signal processing in general, comb filtering approaches represent an important class of filters that play a relevant role in different fields, such as extraction or elimination of periodic signal and harmonic components, speech and audio signal processing, decimation processes, prediction and estimation of geophysical signals, and power line rejection [1–5]. In its simplest form, a comb filter can be viewed as a combination of notch filters in which the null frequencies occur periodically across the filter bandwidth. Another very popular comb filtering approach is the conventional FIR moving average filter indicated in

(1) $$\begin{array}{c} y_{n}=\frac{1}{M}\overset{M-1}{\underset{k=0}{\sum }}x_{n-k}, \end{array}$$

whose representation in *z*-domain and discrete time realisation is shown, respectively, in (2) and Figure 1.

(2) $$\begin{array}{c} \frac{Y\left(z\right)}{X\left(z\right)}=\frac{1}{M}\frac{\left(1-z^{-M}\right)}{\left(1-z^{-1}\right)}=H_{MAF}\left(z\right), \end{array}$$

with *M* = *f*_*s*_/*f*_*M*_, where *f*_*s*_ is the sampling frequency and *f*_*M*_ is the fundamental of the periodic null frequencies.

**Figure 2:**
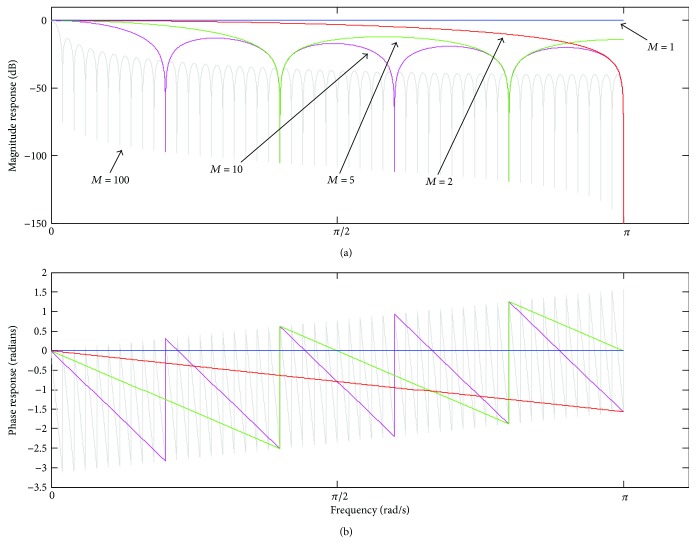
Frequency response of *H*_MAF_(*ω*) for some values of *M*: (a) magnitude response; (b) phase response.

The comb filter realisation indicated in (2) is widely employed because of its computational efficiency. As limitations, however, (2) provides a magnitude response with low attenuation in the filter stopbands as well as nonuniform gain and high attenuation in the pass-bands, as depicted in Figure 2(a). In addition, despite the piecewise linearity of the phase characteristic (Figure 2(b)), it can provoke increased phase delay for higher values of *M* [1, 4].

Such characteristics are undesirable in some applications and are far from those of an ideal comb filter: zero gain at notch frequencies, uniform and unity gain in the pass-bands, and no effects on the signal phase. To make the comb filter realisation of (2) more selective or closer to the ideal behaviour, some strategies have been suggested in the literature. For instance, it can be achieved by the introduction of poles in the transfer function of (2), as indicated in [4, 6, 7]

(3) $$\begin{array}{c} H_{MOD}\left(z\right)=\frac{1-z^{-M}}{1-z^{-1}}\frac{1-r\cdot z^{-1}}{1-{\left(r\cdot z^{-1}\right)}^{M}}, \end{array}$$

where the value of the parameter *r* is contained in the interval 0 ≤ *r* < 1. As mentioned by Proakis and Manolakis [4], the insertion of poles in (2) has the effect of introducing a resonance in the vicinity of the null, thus provoking reduction of the bandwidth of the stopbands. Thereby, the zeros *z* = *e* ^*j*2*πk*/*M*^ placed at the unit circle in the *z*-plane will have in their vicinity the poles *z* = *r* · *e* ^*j*2*πk*/*M*^. The comb filter described in (3) has been successfully used in several applications, such as in harmonic compensators and rectifiers in power systems [6, 7]. However, one of the limitations of this approach is the decreased attenuation in the stopbands, as the value of *r* increases towards 1. Furthermore, there is a trade-off between the values of *M* and *r*, which is contingent to the performance requirement of the filter: on one hand, the use of higher values of *M* makes this method computationally expensive regarding memory usage. On the other hand, the exponential decrease of the power *r* ^*M*^ makes the filter to be implemented by using lower resolution computer unit. Thus, there exists a compromise between the value of *M* and the computer unit resolution [6].

Another proposed strategy to improve the selectivity of the comb filtering provided by (2) is the time-domain averaging approach. Time-domain averaging consists of a kind of comb filtering approach based on a coherent detection process whereby estimation and elimination of the periodic activity are carried out by averaging repetitive sequences of a periodic signal, *p*(*t*), observed in the input signal, *x*(*t*).

(4) $$\begin{array}{c} x\left(t\right)=p\left(t\right)+e\left(t\right). \end{array}$$

In (4), *e*(*t*) represents the nonperiodic component of *x*(*t*), which could be a noise signal or some stochastic process. Under the assumption that *p*(*t*) and *e*(*t*) are uncorrelated, summing up *N* subsequent segments *x*(*t*_*i*_) corresponding to the periodic signal results in coherent summation of *p*(*t*) [8]. Thus, the estimate of *p*(*t*) obtained by time-domain averaging can be calculated by the following discrete representation:

(5) $$\begin{array}{c} {\hat{p}}_{n}=\frac{1}{N}\overset{N-1}{\underset{i=0}{\sum }}x_{n-iM}. \end{array}$$

Or in *z*-domain,

(6) $$\begin{array}{c} H_{p}\left(z\right)=\frac{1}{N}\frac{\left(1-z^{-NM}\right)}{\left(1-z^{-M}\right)}. \end{array}$$

The frequency response associated with the nonperiodic component is derived from the subtraction between the discrete representation of *x*(*t*) and (5).

(7) $$\begin{array}{c} e\left(t\right)=x\left(t\right)-p\left(t\right)\Rightarrow {\hat{e}}_{n}=x_{n}-\frac{1}{N}\overset{N-1}{\underset{i=0}{\sum }}x_{n-iM}=y_{n}. \end{array}$$

(8) $$\begin{array}{c} \Rightarrow \frac{Y\left(z\right)}{X\left(z\right)}=1-H_{p}\left(z\right)\Rightarrow H_{TDA}\left(z\right)=1-\left|H_{p}\left(z\right)\right|, \end{array}$$

where *H*_TDA_(*z*) is the magnitude response of the comb filtering for elimination of the periodic component.

Time-domain averaging is a well-established comb filtering approach which has been widely used to estimate and extract periodic signals encountered in phenomena involving some rotating machinery [2, 8, 9]. Time-domain averaging-based approaches have been also proposed to estimate and eliminate the gradient artefact from the EEG signal, such as the average artefact subtraction method [10, 11]. The gradient artefact consists of a periodic waveform voltage interference which is induced in the electrical potential recorded in the human scalp (scalp potential) by the rapidly varying magnetic field gradients and radiofrequency pulses used in MRI sequences during simultaneous acquisition of EEG and fMRI data [12, 13]. One limitation of time-domain averaging, however, is its high dependency on accurate sampling of the periodic waveform *p*(*t*). The occurrence of jitter errors may result in imprecise sampling of the averaging waveforms, which can impair the effectiveness of the method. Thus, the period of the repetitive waveform must be an exact multiple of the sampling interval. In parallel, the period of *p*(*t*) must be precisely known, which requires an external trigger or reference signal provided by an additional hardware [8, 14]. In case of the suppression of the gradient artefact from the EEG signal, subject movements or small drifts may also compromise the performance of the algorithm, since they change the morphology and shape of the artefact, in such a way that it is not possible to obtain an accurate estimate of $\hat{p}\left(t\right)$. Subject movements or small drifts also provoke broadening of the harmonic artefact spectral lines [15], whose attenuation may not be effectively accounted for by the time-domain averaging comb filter. As a consequence, residual artefacts are left behind in the corrected EEG after subtraction of the estimated periodic waveform ${\hat{p}}_{n}$.

In this paper, we present a novel comb filtering implementation to improve the selectivity of the comb filtering provided by the FIR moving average filter of (2). As described in Section 2, implementation of such a comb filter has been based upon an iterative filtering decomposition process [16], whereby an estimation of the filtered signal can be obtained by the iterative application of a FIR moving average filter-based approach named double average filter. Comparison between the novel comb filtering implementation and those existing methodologies to enhance the selectivity of (2) described above shows that the novel method could be used in scenarios in which those approaches are not effective, such as during broadening of the harmonic gradient artefact spectral lines. In addition, the iterative application of time-domain averaging is revealed to enable the use of a smaller number of averages during application of such a method, as shown in Sections 3 and 4.

## 2. Methods

In recent research [16, 17], iterative filtering decomposition has been proposed as an alternative implementation for empirical mode decomposition [18]. According to this methodology, a series **L**_**i**_ of low-pass filters (or moving average filters) is used to decompose a signal in intrawave frequency modes or intrinsic mode functions (IMFs). Here, we have further exploited the estimation of the first IMF, **F**_1_, by application of the filter (1 − **L**_1_) in the input signal **x**:

(9) $$\begin{array}{c} F_{1}=\lim_{j\to \infty }{\left(1-L_{1}\right)}^{j-1}x, \end{array}$$

where **L**_1_ corresponds to a FIR moving average-based filter. The convergence of the iterative filtering decomposition is ensured by the coefficients (masks) of the filter **L**_1_ having a value between 0 and 1, which has been demonstrated by Lin et al. [16].

### 2.1. Design of a Novel Comb Filtering Approach for Elimination of Periodic Waveforms

As **L**_1_, initially, we investigated the forward-backward application of the moving average filter indicated in (1) in the input signal, *x*_*n*_ [19, 20]. This procedure allows obtaining a filtered signal with zero-phase distortion, which is a characteristic of an ideal comb filter. The forward-backward application of (1) in *x*_*n*_ can be expressed as

(10) $$\begin{array}{c} y_{n}=\frac{1}{M}\overset{0}{\underset{k=M-1}{\sum }}{\left[\frac{1}{M}\overset{M-1}{\underset{k=0}{\sum }}x_{n-k}\right]}_{n+k}=\frac{1}{M}\overset{M-1}{\underset{k=-M+1}{\sum }}\left(\frac{M-\left|k\right|}{M}\right)x_{n+k}. \end{array}$$

**Figure 3:**
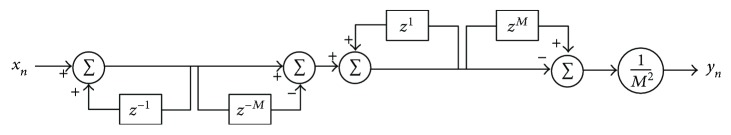
Discrete time realisation of (11).

Equation (10) is also referred to as double average filter [16, 17], where the coefficients of *x*_*n*+*k*_ correspond to a triangular window of length 2 × *M*. By applying the *z*-transformation in (10), it results in the following transfer function:

(11) $$\begin{array}{c} H_{D}\left(z\right)=\frac{1}{{\left(M\right)}^{2}}\frac{\left(1-z^{-M}\right)\left(1-z^{M}\right)}{\left(1-z^{-1}\right)\left(1-z\right)}, \end{array}$$

whose discrete time realisation is depicted in Figure 3.

The frequency response of *H*_*D*_(*z*) is derived from (11) by setting *z* = *e* ^*jω*^. Hence,

(12) $$\begin{array}{c} H_{D}\left(\omega \right)=\frac{1}{{\left(M\right)}^{2}}\frac{\sin^{2}\left(\omega M/2\right)}{\sin^{2}\left(\omega /2\right)}. \end{array}$$

**Figure 4:**
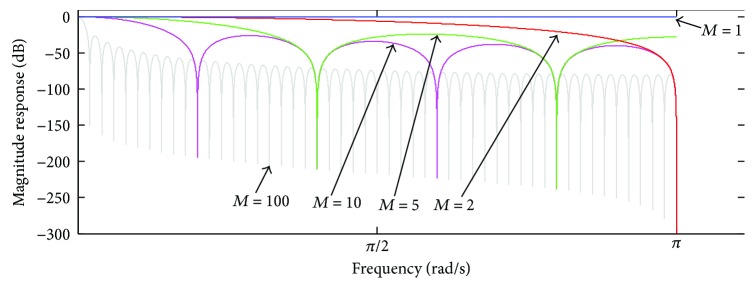
Magnitude response of *H*_*D*_(*ω*) for some values of *M*.

Figure 4 depicts the magnitude response of *H*_*D*_(*ω*), calculated according to (12), for some values of *M*. It also shows the presence of spaced zeros at the frequency 2*π*/*M*. For a hypothetical value *M* = 1, *H*_*D*_(*ω*) becomes an all-pass band filter.

The phase response of *H*_*D*_(*ω*) possesses a zero-phase characteristic, as a result of the forward-backward application of the moving average filter of (1).

(13) θDω=tan−1ImHDωReHDω=0.

Therefore, (11) describes a kind of FIR moving average-based filter that provides no distortion effects in the phase of the signal in the whole filter pass-band. Replacing (11) by **L**_1_ in (9) and taking into account a number *J* of iterations, it can be rewritten as [19]

(14) $$\begin{array}{c} F_{1}\left(z\right)={\left(1-H_{D}\left(z\right)\right)}^{J}X\left(z\right)\Rightarrow \frac{F_{1}\left(z\right)}{X\left(z\right)}={\left(1-H_{D}\left(z\right)\right)}^{J}=H_{1}\left(z\right). \end{array}$$

Equation (14) corresponds to the transfer function that relates the extracted periodic waveform, *p*(*t*), and the input signal, *x*(*t*), as likewise indicated in (4). Therefore, after elimination of *p*(*t*), the output *y*(*t*) of the proposed comb filter has been related to *x*(*t*) as [20]

(15) $$\begin{array}{c} Y\left(z\right)=\left(1-H_{1}\left(z\right)\right)X\left(z\right)\Rightarrow \frac{Y\left(z\right)}{X\left(z\right)}=1-H_{1}\left(z\right)=H_{C}\left(z\right). \end{array}$$

In order to improve the attenuation in the stopbands, we investigated the application of *H*_*C*_(*z*) within the cascade implementation indicated in

(16) $$\begin{array}{c} H_{L}\left(z\right)={\left[H_{C}\left(z\right)\right]}^{L}, \end{array}$$

where *L* is the number of cascades. Since (15) and (16) have been derived from (11), which has zero-phase distortion characteristic, they do not cause any distortion effects on the filtered signal phase either.

### 2.2. Iterative Application of Time-Domain Averaging

As an alternative for the filter **L**_1_ in (9), we have also investigated the use of the time-domain averaging filter described in (8). To this end, we have taken into account a number *J* of iterations of (9), which has been rewritten as

(17) $$\begin{array}{c} F_{2}\left(z\right)={\left(1-H_{TDA}\left(z\right)\right)}^{J}X\left(z\right)\Rightarrow \frac{F_{2}\left(z\right)}{X\left(z\right)}={\left(1-H_{TDA}\left(z\right)\right)}^{J}={\left(H_{p}\left(z\right)\right)}^{J}=H_{2}\left(z\right). \end{array}$$

Thereby, by eliminating the estimated periodic signal, the output has been related to the input as

(18) $$\begin{array}{c} \frac{Y\left(z\right)}{X\left(z\right)}=1-H_{2}\left(z\right)=H_{RTDA}\left(z\right). \end{array}$$

### 2.3. Using the Proposed Method to Design a Novel Notch Filtering Approach

As remarked by Braun [2], the frequency response provided by (7) and (8) corresponds to the convolution between the frequency response depicted in Figure 2 and a train of unit pulses separated by the period *M*. Making use of this idea, we also investigated the convolution of a single pulse, δ, and the magnitude response indicated in Figure 2(a) to design a novel notch filtering approach approximated to the ideal case.

(19) $$\begin{array}{c} H_{3}\left(\omega \right)=\delta \left(\omega \right)*\left|H_{MAF}\left(\omega \right)\right|. \end{array}$$

**Figure 5:**
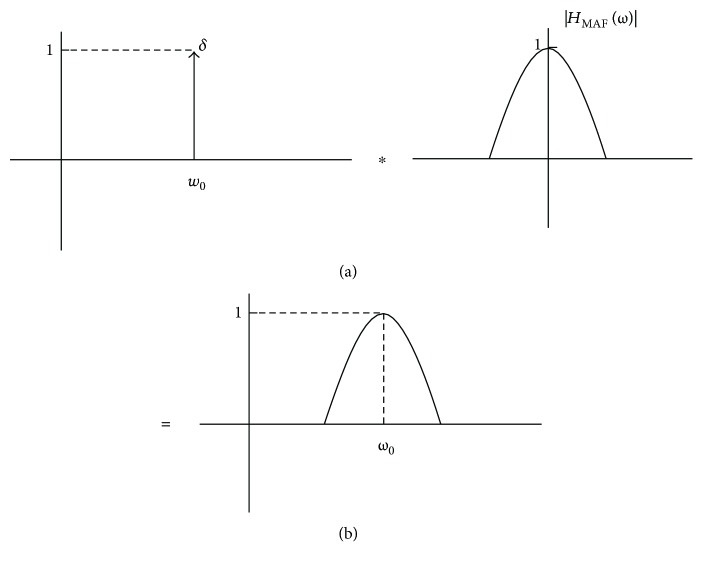
Convolution between the unit pulse *δ* (located at the frequency *ω*_0_) and |*H*_MAF_(*ω*)| (*M* = 2). It results in a pass-band filter with a central frequency at *ω*_0_.

Figure 5 shows this convolution, where the unit pulse has been located at the frequency *ω*_0_, and |*H*_MAF_(*ω*)| was calculated for *M* = 2.

As can be noticed in Figure 5(b), (19) corresponds to a pass-band filter with unit amplitude and central frequency at *ω*_0_. Replacing **L**_1_ in (9) by (1 − *H*_3_(*z*)) and taking into account a certain number *J* of iterations, it results in the notch filter *H*_NTC_(*z*) of

(20) $$\begin{array}{c} F_{3}\left(z\right)={\left[1-\left(1-H_{3}\left(z\right)\right)\right]}^{J}X\left(z\right)\Rightarrow \frac{F_{3}\left(z\right)}{X\left(z\right)}={\left(H_{3}\left(z\right)\right)}^{J}\Rightarrow H_{NTC}\left(z\right)=1-{\left(H_{3}\left(z\right)\right)}^{J}. \end{array}$$

Like in (16), we investigated the application of (20) in a cascade implementation to enlarge the attenuation at the notch frequency, as indicated in

(21) $$\begin{array}{c} H_{NL}\left(z\right)={\left[H_{NTC}\left(z\right)\right]}^{L}, \end{array}$$

where *L* represents the number of cascades as well.

## 3. Results

### 3.1. Frequency Characteristics of the Novel Comb Filtering Approach

All frequency responses depicted below have been calculated using a number of samples *N*_*s*_ = 300000 samples, so that *ω* was set as *ω* = [−*π*, *π*], with a frequency interval at 2*π* × (1/*N_s_*). In these figures, only the frequencies ranging from 0 to *π* are shown. The simulations were performed in MATLAB environment (The MathWorks Inc., Natick, USA).

**Figure 6:**
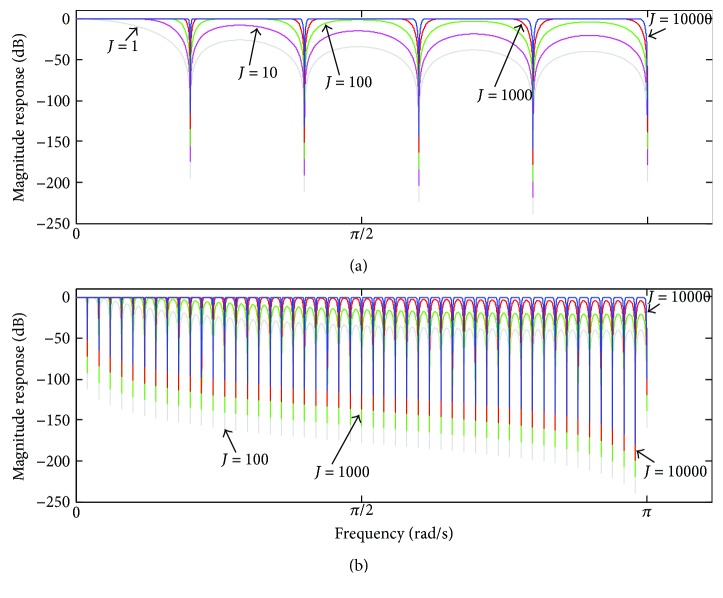
Magnitude response of *H*_*C*_(*ω*), taking into account (a) *M* = 10 and (b) *M* = 100 and some values of *J*.

**Figure 7:**
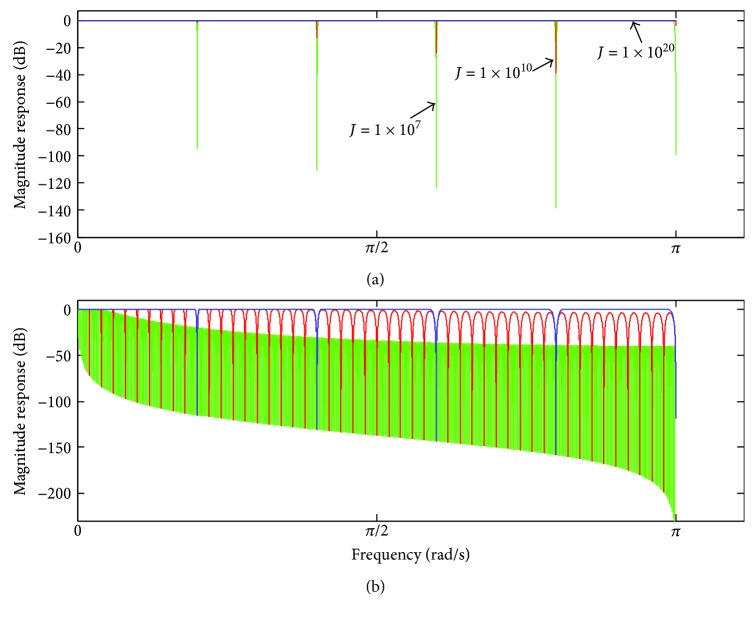
(a) Magnitude response of *H*_*C*_(*ω*) for *M* = 10 and some values of *J* (*J* → ∞); (b) magnitude response of *H*_*C*_ for *J* = 10000 and some values of *M*: *M* = 10 (blue trace), *M* = 100 (red trace), and *M* = 1000 (green trace).

In Figure 6, the magnitude response of *H*_*C*_(*z*) (15) is depicted, taking into account *M* = 10 and *M* = 100 and some values of *J*. It can be observed that increasing of *J* is followed by substantial increasing of the filter gain, which attained 0 dB as well as became more uniform in the different pass-bands. On the other hand, increasing of *J* is also followed by a reduction in the attenuation in the filter stopbands. On the extreme case, when *J* → ∞, (15) tends to become an all-pass band filter, as shown in Figure 7(a). By fixing the value of *J* and varying *M*, (15) shows to provide more uniform gain in the pass-bands for smaller values of *M*, as observed in Figure 7(b).

**Figure 8:**
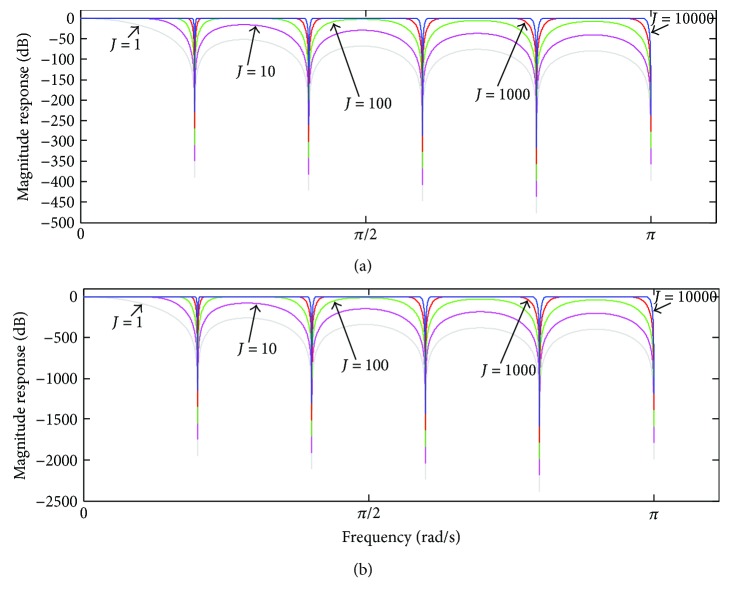
Magnitude response of *H*_*L*_(*ω*) (16) for (a) *L* = 2 and (b) *L* = 10, taking into account *M* = 10 and some values of *J*.

To demonstrate the enhancement of attenuation in the stopbands using (16), its magnitude response has been calculated taking into consideration different values of *L*.  Figure 8 depicts the magnitude response of *H*_*L*_(*z*), for *L* = 2 and *L* = 10, and taking into account *M* = 10, and some values of *J*. As can be noticed, (16) provides higher attenuation in the stopbands by increasing the value of *L*.

### 3.2. Iterative Application of Time-Domain Averaging

By setting *z* = *e* ^*jω*^ in (6), the magnitude and phase response of the periodic component $\hat{p}\left(t\right)$ obtained by time-domain averaging can be, respectively, derived as

(22) $$\begin{array}{c} \left|H_{p}\left(\omega \right)\right|=\frac{1}{N}\left|\frac{\sin\left(\omega NM/2\right)}{\sin\left(\omega M/2\right)}\right| \end{array}$$

and

(23) θpω=tan−1ImHpωReHpω.

**Figure 9:**
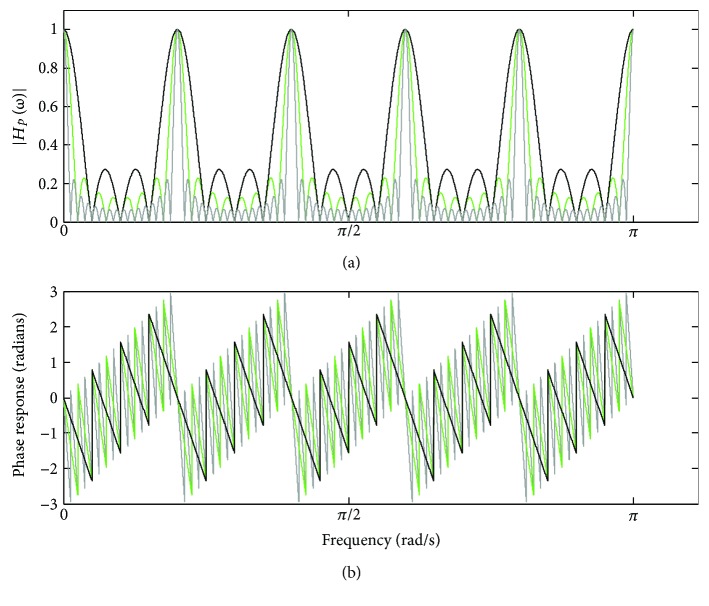
Frequency response of *H*_*p*_(*ω*) for *M* = 10, taking into account *N* = 4 (dark trace), *N* = 8 (green trace), and *N* = 16 (gray trace): (a) magnitude response; (b) phase response.

Figure 9 depicts the magnitude and phase response of (22) and (23), taking into account *M* = 10, for *N* = 4 (dark trace), *N* = 8 (green trace), and *N* = 16 (gray trace).

**Figure 10 fig10:**
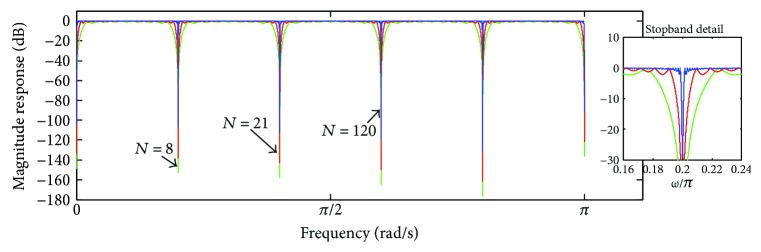
Magnitude response of *H*_TDA_(*ω*) ((8) for *M* = 10 and some values of *N*.

The main lobes of the magnitude response |*H*_*p*_(*ω*)| lie spaced at the frequency 2*π*/*M* and have unit amplitude. By increasing *N*, the amplitude of the side lobes is reduced as well as the main lobes are narrowed. Regarding the phase response, it is not linear, but has approximated piecewise linearity in between the null frequencies of the lobes [4, 8, 9]. In turn, Figure 10 shows the frequency response associated with the nonperiodic component *e*(*t*), as indicated in (8).

**Figure 11 fig11:**
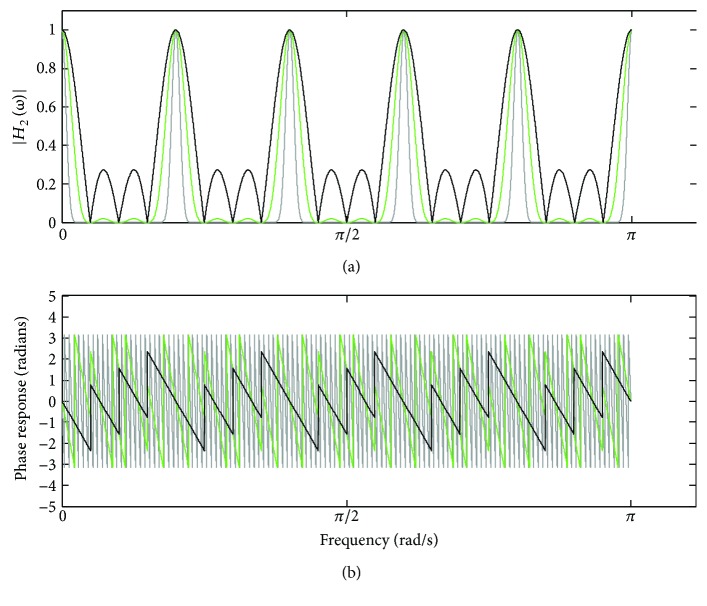
Frequency response of (17), taking into account *M* = 10 and *N* = 4, for *J* = 1 (dark trace), *J* = 3 (green trace), and *J* = 8 (gray trace): (a) magnitude response; (b) phase response.

Figure 11 depicts the frequency response provided by (17), taking into account *M* = 10 and *N* = 4, for *J* = 1 (dark trace), *J* = 3 (green trace), and *J* = 8 (gray trace).

**Figure 12:**
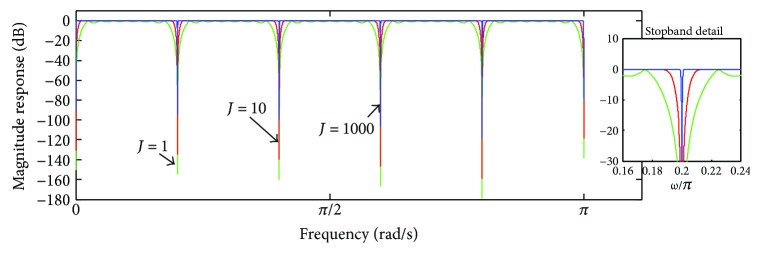
Magnitude response of *H*_RTDA_(*ω*) ((18), taking into account *M* = 10 and *N* = 8 and some values of *J*.

It can be noticed that increasing *J* in (17) has a similar effect to increasing *N* in (22), by reducing the amplitude of the side lobes and narrowing the main lobes. Regarding the phase response, it remains piecewise linear after increasing *J*. In Figure 12, the frequency response corresponding to (18) is depicted, for *M* = 10, *N* = 8, and some values of *J*. It can be observed that as *J* increases, the filter gain is approximated to unity along the pass-bands as well as the stopbands become narrower. Therefore, an increase of *J* in (18) attests a similar effect to that provided by increasing *N* in (8) alike.

### 3.3. Notch Filter Characteristics Obtained by Iterative Filtering

**Figure 13:**
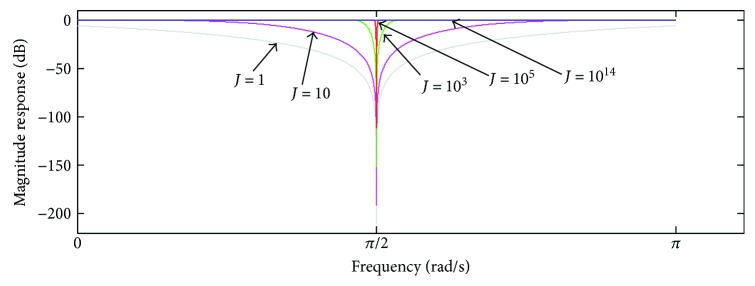
Magnitude response of *H*_NTC_(*ω*) ((20), taking into account some values of *J* and cutoff frequency at *ω*_0_ = *π*/2.

The magnitude response corresponding to *H*_NTC_(*z*) is depicted in Figure 13, taking into account *M* = 2, some values of *J*, and *N*_*s*_ = 300000 samples for |*H*_MAF_(*ω*)| as well. The notch filter stopband was located at *ω*_0_ = *π*/2. It can be observed that the bandwidth of the notch filter stopband is narrowed by increasing the value of *J*, as well as (20) tends to become an all-pass band filter when *J* → ∞. This notch filter does not cause any effects on the phase of the signal.

**Figure 14:**
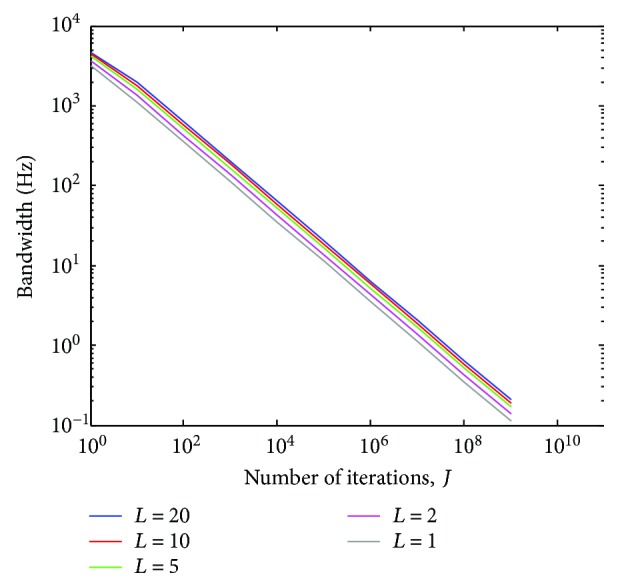
Bandwidth of the notch filter stopband around −3 dB ((21), taking into account *M* = 2 and some values of *J* and *L*. *f*_*s*_ = 5 kHz.

In Figure 14, we show the use of (20) within the cascade implementation indicated in (21). The variation of the bandwidth of the notch filter stopband around −3 dB provided by (21) is indicated, taking into account *M* = 2, some values of *J* and *L*, *f* = (*ω*/2*π*) × *f*_*s*_, and *f*_*s*_ = 5 kHz. As can be seen, combination of proper values of *J* and *L* allows obtaining stopband bandwidths ranging from 0 (when *J* → ∞) up to 4500 Hz (for *L* = 20). Therefore, (21) can produce a notch filter with a large range of values for the stopband bandwidth around −3 dB.

## 4. Further Comparative Analyses and Discussion

**Figure 15:**
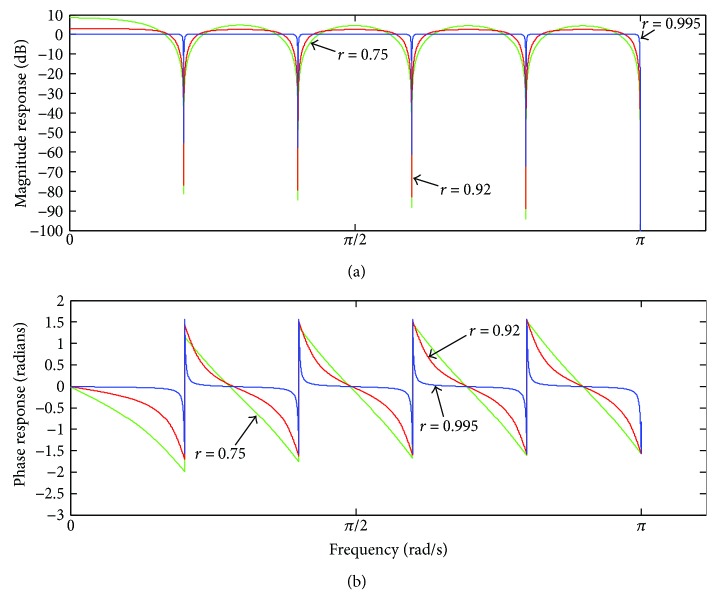
Frequency response of *H*_MOD_(*ω*) for some values of *r*: (a) magnitude response; (b) phase response.

Figure 15 shows the frequency response of (3), for some values of *r* and *M* = 10.

It can be noticed that as the value of *r* increases towards 1, the gain of the filter approximates to unity and becomes more uniform along the different pass-bands, as well as the phase response approaches to a zero-phase distortion. On the extreme scenario of *r* → 1, the filter becomes an all-pass band filter. On the other extreme, when *r* = 0, (3) equals (2) [6].

As indicated in Figures 6 and 10, by increasing the parameters *J* ((15) and *N* ((8), the gain in the filter pass-bands provided, respectively, by *H*_*C*_ and *H*_TDA_ increases and attains unity (0 dB). Simultaneously, a reduction of attenuation in the stopbands is also observed. Nevertheless, rather than the response of *H*_MOD_ shown in Figure 15, there is no effect on the phase response corresponding to *H*_*C*_ and *H*_TDA_, which remains zero radians for any values of frequency. On the other hand, *H*_TDA_ ((8) cannot be applied in a cascade implementation because of the ripple that occurs below and around 0 dB in the pass-bands (see stopband detail in Figure 10), which can compromise the gain uniformity along the pass-bands. As *H*_*C*_ shows no ripple along the pass-bands and no phase distortion, these characteristics allow the application of *H*_*C*_ in a cascade implementation ((15) to improve the attenuation in the stopbands. *H*_MOD_, in turn, cannot be applied within a cascade implementation without causing some distortion in the signal phase alike.

**Figure 16:**
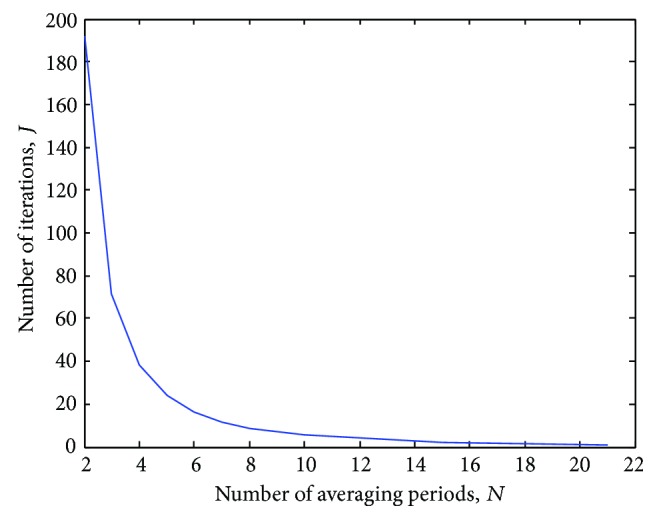
Variation of the number of averaging epochs (*N*) according to the number of iterations (*J*) in (18), taking into account a certain bandwidth (18.01 Hz) in the pass-bands around −3 dB. *f*_*s*_ = 5 kHz.

Figures 9 [Fig fig10] [Fig fig11]–12 reveal that the iterative application of time-domain averaging, according to (18), represents an alternative to using higher values of *N*, since it permits to obtain narrower comb filter stopbands as well as phase distortion approximately zero along the filter pass-bands. Thus, in addition to enhancing the comb filter selectivity, time-domain averaging applied with a *J* number of iterations enables to use a smaller number of averaging periods. In Figure 16, we illustrate the variation of the number of averaging epochs according to the number of iterations, which was calculated by taking into consideration a certain bandwidth (18.01 Hz) in the stopbands around −3 dB and *M* = 20.

For instance, by using *J* = 1 and *N* = 21 averaging epochs, it was possible to produce the same bandwidth in the stopbands around −3 dB by setting *N* = 3 and *J* = 71.77 in (18). Therefore, when *J* ≠ 1 in (18), the attenuation of the noise component is carried out by a factor higher than $\sqrt{N}$ [8].

**Figure 17:**
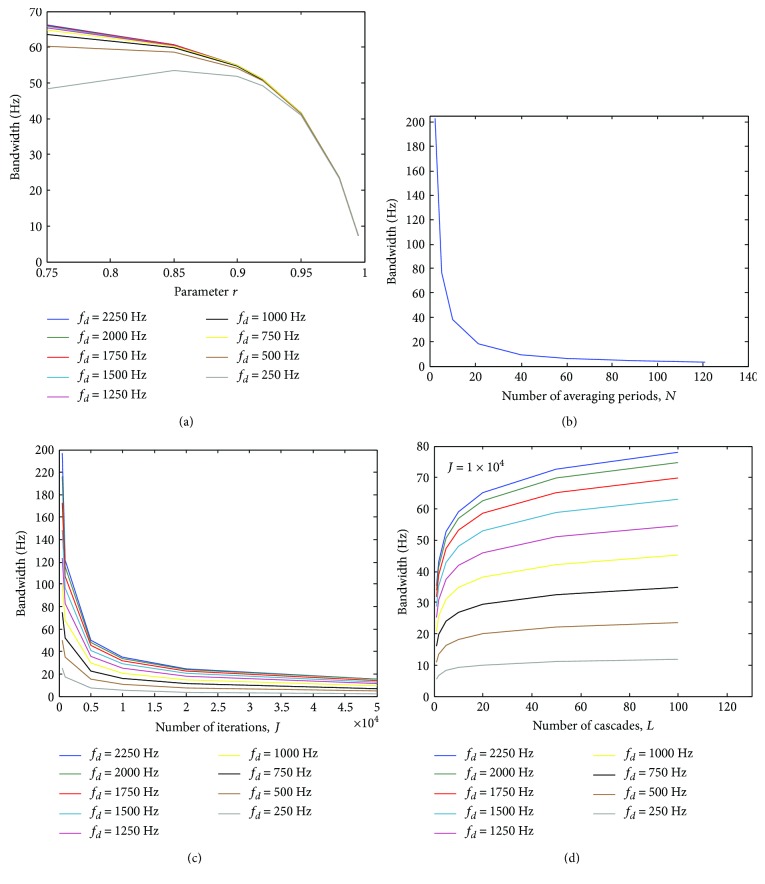
Bandwidth of the stopbands around −3 dB (*M* = 20), for (a) *H*_MOD_, (b) *H*_TDA_, (c) *H*_*C*_, and (d) *H*_*L*_ (*J* = 1 × 10^4^). *f*_*s*_ = 5 kHz.

Figure 17 shows the difference amongst *H*_MOD_, *H*_TDA_, *H*_*C*_, and *H*_*L*_ regarding the bandwidth in the stopbands around −3 dB.

The analysis depicted in this figure has taken into account *M* = 20. In case of *H*_MOD_, above around *r* = 0.95, the bandwidth around −3 dB is equal for all stopbands. In turn, for *H*_TDA_ (and *H*_RTDA_), such a bandwidth is approximately similar for all stopbands, irrespective of the value of *N* (and *J*). On the other hand, for *H*_*C*_, the width of the stopbands around −3 dB depends on the frequency of the stopband as well as the value of *J*. The attenuation in the stopbands (as well as the gain in the pass-bands) may lie below −3 dB for smaller values of *J* (see Figure 6). As the gain in the pass-bands approximates to 0 dB when *J* increases, the bandwidth of the stopbands around −3 dB becomes narrower. However, it is not the same for all stopbands, but it enlarges according to the fundamental frequency (*f_d_*) of the stopband (Figure 17(c)). Such a difference is even higher for smaller values of *J*. Therefore, as shown in Figure 17(c), the higher the fundamental frequency of the stopband, the broader is its respective bandwidth. This characteristic has been demonstrated to be useful for obtaining a more effective attenuation in the frequency bins associated with the harmonic gradient artefact spectral lines, with similar preservation of the EEG signal than that provided by the AAS method which is based upon time-domain averaging [20]. Thereby, broadening of the gradient artefact spectral lines provoked by micromovements of the subject head with the fMRI scanner, which are mostly observed in higher frequencies, can be more effectively accounted for by *H*_*C*_ because of the increasing stopband bandwidths around −3 dB according to *f_d_*.

**Figure 18:**
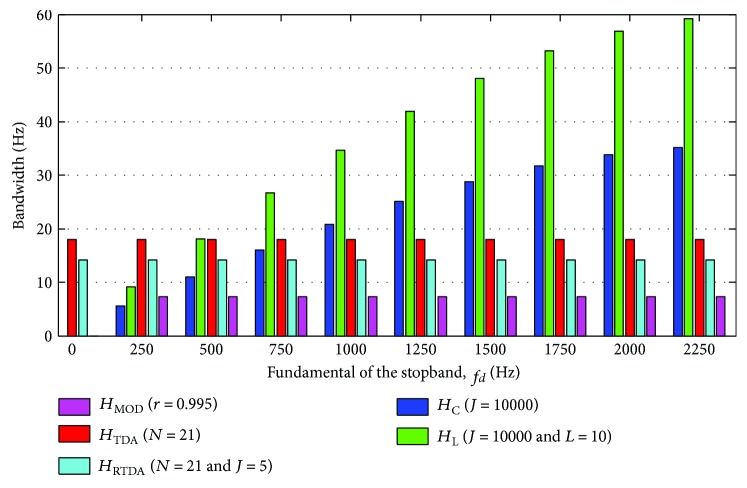
Bandwidth of the stopbands around −3 dB (*M* = 20) for an exemplary setting of parameters *r*, *N*, *J*, and *L*. *f*_*s*_ = 5 kHz.

In Figure 18, an exemplary setting of parameters *r*, *N*, *J*, and *L* are used to illustrate the bandwidth of the stopbands according to the fundamental frequency (*f_d_*). As predicted by (16), the stopband bandwidth is further enlarged when *H*_*C*_ is applied within the cascade implementation (*H*_*L*_), especially in stopbands with higher fundamental frequency (Figures 17(d) and 18). As can also be observed in Figure 18, *H*_*C*_, *H*_*L*_, and *H*_MOD_ do not provide a stopband around 0 Hz, whereas it is produced by *H*_TDA_ and *H*_RTDA_.

**Figure 19:**
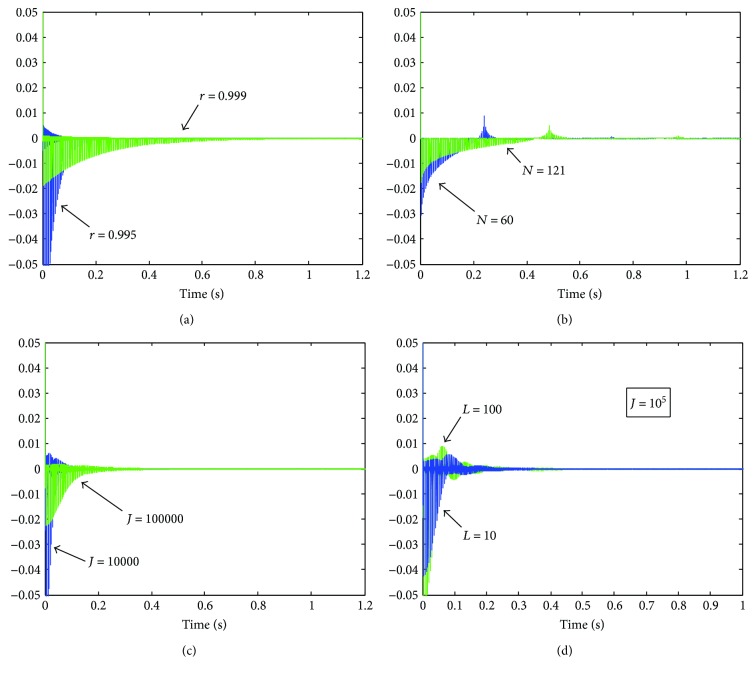
Impulse response (*M* = 20), for (a) *H*_MOD_, (b) *H*_TDA_, (c) *H*_*C*_, and (d) *H*_*L*_ (*J* = 10^5^).

Figure 19 depicts the impulse response associated with *H*_MOD_, *H*_TDA_, *H*_*C*_, and *H*_*L*_, for some values of the parameters *r*, *N*, *J*, and *L*. After the impulse is applied, an overshoot occurs in each of these responses, followed by decaying peaks spaced at the window length *M*. The peak overshoot as well as the duration of the decaying peaks depend on the value of the parameter: smaller *r*, *N*, and *J* produce an increase in the overshoot and decreased settling time. Rather, a higher value of *L* provides an increase in the overshoot and increased settling time.

Regarding the computational effort, we verified that *H*_*C*_ and *H*_*L*_ are less computationally demanding than *H*_MOD_, *H*_TDA_, and *H*_RTDA_. This can be ascribed to the computational efficiency of (11), which precisely requires the double of the computations of (2), as indicated in Figures 1 and 3. The worst case of computational effort was for *H*_MOD_, whose application was seriously compromised because of expensive computational memory demand by higher values of *M* [6, 7].

Since a comb filter can be also implemented as a combination of notch filters, the selectivity provided by (21) could be used to design a highly selective comb filtering approach with a variable bandwidth for the different filter stopbands. Regarding the use of values of *M* > 2 in (20), we observed that some ripple around and below 0 dB may appear in the frequency response of *H*_NTC_ for smaller values of *J*, which can cause nonuniform gain along the pass-bands within the cascade implementation indicated in (21). Case higher values of *J* are used in this scenario, the filter gain becomes uniform, but the notch filter stopband would have narrower bandwidth than *M* = 2 in (21).

In future work, (20) should have its performance compared with other notch filter approaches [21], as well as (21) should be used and evaluated in applications where notch or comb filtering is required, such as power line rejection in biomedical signals [1]. Additionally, other filters **L**_1_ in (9) should be investigated and evaluated for the iterative comb filtering implementation proposed here. Equations (15) and (16) should also be used and have their performance evaluated in signal processing applications other than the suppression of the gradient artefact from the EEG recordings [19, 20], where broadening of harmonic spectral lines is observed. As a further suggestion for future work, the iterative application of time-domain averaging should be assessed by using other kinds of moving-averaging filters, such as the exponential averaging and running averaging [2]. Last, the novel comb and notch filtering implementation described in this work shall be used and evaluated in other biomedical signal processing applications, such as speech signal processing and during estimation of evoked potentials (EPs) and event-related potential (ERPs) responses [1, 22].

## 5. Conclusions

A number of biomedical and other signal processing applications require the use of comb filtering approaches that perform elimination or extraction of periodic waveforms with a high degree of selectivity. As regards the elimination of periodic waveforms, the comb filter should be able to suppress the harmonics associated with the periodic signal and, simultaneously, to preserve the stochastic component or noisy signal according to the level of quality required by the application. Thus, it is important to make sure that the performance of the comb filtering approach meets the selectivity requirements of the application. Since such requirements are not always met by the existing methods, investigation and proposal of novel approaches to improve the comb filtering selectivity have been often described in the literature.

In this work, we have demonstrated how iterative filtering can be used to improve the selectivity of comb filtering approaches as well as to design a novel notch filter, which are based on the conventional FIR moving average filter. The novel comb filtering approach implementation is revealed to provide unity gain in the pass-bands, no effects on the signal phase, and broadening of the stopband bandwidth around −3 dB according to the fundamental frequency of the stopband. This characteristic has been proven to be useful within a scenario of broadening of spectral lines, such as that observed during the occurrence of the gradient artefact in the EEG signal recorded simultaneously with fMRI data. Moreover, a cascade implementation of the proposed approach permits to further increase the attenuation provided in the stopbands. In parallel, the iterative application of time-domain averaging allows using a smaller number of averaging epochs in order to estimate the periodic signal component. Hence, when a number of iterations are taken into consideration, the noise component can be attenuated with a factor higher than the square roots of the number of averaging epochs. Last, the novel notch filter implementation by iterative decomposition shows to provide a selective filtering with a large range of values for the stopband bandwidth around −3 dB.

## References

[1] Rangayyan R. M. (2002). *Biomedical Signal Analysis: A Case-Study Approach*.

[2] Braun S. (2011). The synchronous (time domain) average revisited. *Mechanical Systems and Signal Processing*.

[3] Meher J., Meher P., Dash G. (2011). Improved comb filter based approach for effective prediction of protein coding regions in DNA sequences. *Journal of Signal and Information Processing*.

[4] Proakis J. G., Manolakis D. G. (1996). *Digital Signal Processing: Principles, Algorithms, and Applications*.

[5] Zeng Z., Xie Y., Wang Y., Guan Y., Li L., Zhang X. An improved harmonic current detection method based on parallel active power filter.

[6] Tahir M., Mazumder S. K. (2014). Improving dynamic response of active harmonic compensator using digital comb filter. *IEEE Journal of Emerging and Selected Topics in Power Electronics*.

[7] Prodić A., Chen J., Maksimović D., Erickson R. W. (2003). Self-tuning digitally controlled low-harmonic rectifier having fast dynamic response. *IEEE Transactions on Power Electronics*.

[8] Braun S. (1975). The extraction of periodic waveforms by time domain averaging. *Acustica*.

[9] McFadden P. D. (1987). A revised model for the extraction of periodic waveforms by time domain averaging. *Mechanical Systems and Signal Processing*.

[10] Allen P. J., Josephs O., Turner R. (2000). A method for removing imaging artifact from continuous EEG recorded during functional MRI. *NeuroImage*.

[11] Becker R., Ritter P., Moosmann M., Villringer A. (2005). Visual evoked potentials recovered from fMRI scan periods. *Human Brain Mapping*.

[12] Anami K., Mori T., Tanaka F. (2003). Stepping stone sampling for retrieving artifact-free electroencephalogram during functional magnetic resonance imaging. *NeuroImage*.

[13] Yan W. X., Mullinger K. J., Brookes M. J., Bowtell R. (2009). Understanding gradient artefacts in simultaneous EEG/fMRI. *NeuroImage*.

[14] Mandelkow H., Halder P., Boesiger P., Brandeis D. (2006). Synchronisation facilitates removal of MRI artefacts from concurrent EEG recordings and increases usable bandwidth. *NeuroImage*.

[15] Spencer G. S. (2015). *EEG-fMRI: Novel Methods for Gradient Artefact Correction [Ph.D. Thesis]*.

[16] Lin L., Wang Y., Zhou H. (2009). Iterative filtering as an alternative algorithm for empirical mode decomposition. *Advances in Adaptive Data Analysis*.

[17] Wang Y., Wei G.-W., Yang S. (2012). Iterative filtering decomposition based on local spectral evolution kernel. *Journal of Scientific Computing*.

[18] Huang N. E., Shen Z., Long S. R. (1998). The empirical mode decomposition and the Hilbert spectrum for nonlinear and non-stationary time series analysis. *Proceedings of the Royal Society A: Mathematical, Physical and Engineering Sciences*.

[19] Ferreira J. L., Aarts R. M., Cluitmans P. J. M. Optimized moving-average filtering for gradient artefact correction during simultaneous EEG-fMRI.

[20] Ferreira J. L., Wu Y., Besseling R. M. H., Lamerichs R., Aarts R. M. (2016). Gradient artefact correction and evaluation of the EEG recorded simultaneously with fMRI data using optimised moving-average. *Journal of Medical Engineering*.

[21] Vlček M., Zahradník P. (2004). Fast analytical design algorithms for FIR notch filters. *IEEE Transactions on Circuits and Systems I: Regular Papers*.

[22] Sanei S., Chambers J. A. (2007). *EEG Signal Processing*.

